# Major exopolysaccharide, EPS I, is associated with the feedback loop in the quorum sensing of *Ralstonia solanacearum* strain OE1‐1

**DOI:** 10.1111/mpp.12870

**Published:** 2019-09-27

**Authors:** Kazusa Hayashi, Wakana Senuma, Kenji Kai, Akinori Kiba, Kouhei Ohnishi, Yasufumi Hikichi

**Affiliations:** ^1^ Faculty of Agriculture and Marine Science Kochi University Nankoku Kochi 783‐8502 Japan; ^2^ Graduate School of Life and Environmental Sciences Osaka Prefecture University Sakai Osaka 599‐8531 Japan; ^3^ Present address: Kochi Prefectural Agriculture Research Center Nankoku Kochi 783‐0023 Japan

**Keywords:** major exopolysaccharide EPS I, quorum sensing, *Ralstonia solanacearum*

## Abstract

The Gram‐negative soil‐borne bacterium *Ralstonia solanacearum* first infects roots of host plants and then invades xylem vessels. In xylem vessels, the bacteria grow vigorously and produce exopolysaccharides (EPSs) to cause a wilt symptom on host plants. The EPSs are thus the main virulence factors of *R. solanacearum*. The strain OE1‐1 of *R. solanacearum* produces methyl 3‐hydroxymyristate as a quorum‐sensing (QS) signal, and senses this QS signal, activating QS. The QS‐activated LysR‐type transcriptional regulator PhcA induces the production of virulence‐related metabolites including ralfuranone and the major EPS, EPS I. To elucidate the function of EPS I, the transcriptomes of *R. solanacearum* strains were analysed using RNA sequencing technology. The expression of 97.2% of the positively QS‐regulated genes was down‐regulated in the *epsB*‐deleted mutant Δ*epsB*, which lost its EPS I productivity. Furthermore, expression of 98.0% of the negatively QS‐regulated genes was up‐regulated in Δ*epsB*. The deficiency to produce EPS I led to a significantly suppressed ralfuranone productivity and significantly enhanced swimming motility, which are suppressed by QS, but did not affect the expression levels of *phcA* and *phcB,* which encode a methyltransferase required for methyl 3‐hydroxymyristate production. Overall, QS‐dependently produced EPS I may be associated with the feedback loop of QS.

The Gram‐negative bacterium *Ralstonia solanacearum* infects more than 250 plant species worldwide and causes bacterial wilt (Mansfield *et al.*, 2012). The soil‐borne *R. solanacearum* first invades plant roots through wounds or natural openings, colonizes the root intercellular spaces and then invades xylem vessels (Araud‐Razou *et al.*, 1998; Hikichi *et al.*, 2017; Vasse *et al.*, 1995). *Ralstonia solanacearum* produces and extracellularly secretes nitrogen‐ and carbohydrate‐rich exopolysaccharides (EPSs) dependent on quorum sensing, which consists of phc regulatory elements (*phc* QS), indicating that EPSs are abundantly produced at high cell densities (McGarvey *et al.*, 1999; Schell, 2000). In xylem vessels, *R. solanacearum* grows vigorously and produces copious EPSs. EPS productivity‐deficient mutants of *R. solanacearum* show significantly reduced virulence levels (Denny and Baek, 1991). It is thus thought that the EPSs are main virulence factors.

Bacterial cells release QS signals, which are small diffusible signal molecules, and monitor QS signals to track changes in their cell numbers (Rutherford and Bassler, 2012). *Ralstonia solancearum* strains AW1 and K60, and OE1‐1 and GMI1000, produce methyl 3‐hydroxypalmitate and methyl 3‐hydroxymyristate (3‐OH MAME), respectively, as QS signals with the methyltransferase PhcB (Flavier *et al.*, 1997; Kai *et al.*, 2015). These QS signals are sensed through the histidine kinase PhcS sensor, leading to the activation of *phc *QS (Fig. S1, see Supporting information). The LysR‐type transcriptional regulator PhcA, activated through *phc *QS, plays a central role among global virulence regulators, including major EPS I (Genin and Denny, 2012).

PhcA, when activated by *phc *QS, also induces the expression of *ralA*, encoding ralfuranone synthase, which induces the production of a precursor, ralfuranone I, for the aryl‐furanone secondary metabolites, ralfuranones (Fig. S1). Ralfuranone B is produced by an extracellular non‐enzymatic conversion from ralfuranone I. The enzymatic oxidation of ralfuranone B produces ralfuranones J and K. The benzaldehyde is non‐enzymatically eliminated from ralfuranone B, producing ralfuranone A (Kai *et al.*, 2014, 2016; Pauly *et al.*, 2013). Interestingly, ralfuranones also affect the function of PhcA in positively feedback‐regulating *phc *QS (Hikichi *et al.*, 2017; Mori *et al.*, 2018b). Furthermore, functional PhcA induces the expression of *lecM*, encoding the lectin LecM (Meng *et al.*, 2015; Mori *et al.*, 2016). LecM affects the extracellular stability of 3‐OH MAME, contributing to the *phc *QS signalling pathway (Hayashi *et al.*, 2019).

EPSs cloak bacterial surface features, which plants can recognize, to protect *R. solanacearum* from plant antimicrobial defences (D’Haeze and Holsters, 2004). Although EPS production is metabolically expensive for *R. solanacearum*, EPSs are involved in the bacterial survival under desiccation conditions in soil (Denny, 1995; McGarvey *et al.*, 1999; Peyraud *et al.*, 2016; Saile *et al.*, 1997). Furthermore, *R. solanacearum* that has infected xylem vessels produces copious amounts of EPS I, which is essential for wilting because it restricts water flow through xylem vessels, killing the hosts (Genin and Denny, 2012). Interestingly, bacterial wilt‐resistant tomato plants specifically recognize EPS I from *R. solanacearum* (Milling *et al.*, 2011), demonstrating that EPS I also functions as a signal for plant–*R. solanacearum* interactions. However, the role of EPS I in the intracellular and intercellular signalling of *R. solanacearum* cells associated with the bacterial virulence has remained unknown.

The *eps* operon containing *epsB*, of which expression is positively regulated by the *phc *QS, is involved in the production and extracellular secretion of EPS I (Genin and Denny, 2012). In this study, to elucidate the functions of EPS I, transcriptome profiles of *R. solanacearum* strains, including the *epsB*‐deletion mutant Δ*epsB* that lost its EPS I productivity (Mori *et al.*, 2018a), were first examined using RNA sequencing (RNA‐Seq) technology. The functions of EPS I in *phc *QS‐dependent *R. solanacearum* virulence‐related phenotypes were then analysed.

We first analysed the transcriptomes of *R. solanacearum* strains, the Δ*epsB* mutant, the *phcB*‐deletion mutant Δ*phcB* (Kai *et al.*, 2015), the *phcA*‐deletion mutant Δ*phcA* (Mori *et al.*, 2016) and the strain OE1‐1 (Kanda *et al.*, 2003), which were cultured in one‐quarter‐strength M63 medium (1/4 M63; Cohen and Rickenberg, 1956) until the optical density at 600 nm (OD_600_) was 0.3. RNA sample preparation and sequencing were performed as previously described (Hayashi *et al*., 2019). RNA‐Seq data mapping and analyses were performed with an Illumina HiSeq 2000 system (Illumina, Madison, WI, USA) according to the protocols of Hayashi *et al*. (2019). For each strain, we conducted two biologically independent experiments. After RNA‐Seq using a final 400 ng RNA yield, the two experiments produced 46.5 and 43.9, 45.3 and 44.4, 45.4 and 44.9, and 47.0 and 46.0 million 100‐bp paired‐end reads from the strains Δ*epsB*, Δ*phcB*, Δ*phcA* and OE1‐1, respectively. An iterative alignment per experiment successfully mapped 42.5 and 43.5, 42.1 and 44.1, 44.7 and 44.5, and 41.8 and 43.8 million 100‐bp paired‐end reads from the strains Δ*epsB*, Δ*phcB*, Δ*phcA* and OE1‐1, respectively, to the reference genome of *R. solanacearum* strain GMI1000 (Salanoubat *et al.*, 2002). We identified 4501 protein‐coding transcripts from the RNA‐Seq reads of strain OE1‐1 by mapping to the GMI1000 genome (Table S1, see Supporting information). The read counts of each sample as fragments per kilobase of open reading frame per million fragments mapped values were calculated with Cufflinks v. 2.2.1 (http://cole-trapnell-lab.github.io/cufflinks/). The normalized gene expression levels of strains Δ*epsB*, Δ*phcB* and Δ*phcA* were compared with that of strain OE1‐1 to detect differentially expressed transcripts. Genes were considered differentially expressed if they exhibited expression level fold‐changes ≤−4 or ≥4 [log_2_(fold changes) ≤−2 or ≥2].

We observed significantly lower expression levels of 411 genes (positively PhcA‐regulated genes) in Δ*phcA* than in strain OE1‐1 (Fig. 1A and Table S1). The expression levels of 407 genes (positively PhcB‐regulated genes) were significantly lower in Δ*phcB*. Among the positively PhcB‐regulated genes, 356 genes were also included in the positively PhcA‐regulated genes, suggesting that these genes are the positively *phc *QS‐regulated genes. The expression levels of 140 genes (negatively PhcA‐regulated genes) in Δ*phcA* were significantly greater than in strain OE1‐1 (Fig. 1B and Table S1). The expression levels of 155 genes (negatively PhcB‐regulated genes) in Δ*phcB* were significantly greater than in strain OE1‐1. Among the negatively PhcB‐regulated genes, 100 genes were included in the negatively PhcA‐regulated genes, suggesting that these genes are the negatively *phc *QS‐regulated genes. We detected 477 genes (EPS I‐positively regulated genes) and 198 genes (EPS I‐negatively regulated genes) that were expressed at significantly lower and higher levels, respectively, in Δ*epsB* than in OE1‐1 (Fig. 1A and Table S1). Among the positively EPS I‐regulated genes, 346 genes were included in the positively *phc *QS‐regulated genes, including not only ralfuranone production‐related genes (i.e. *ralA* and *ralD*), *lecM*, plant cell wall degradation enzyme genes (i.e. *cbhA*, *egl* and *pme*), the effector gene *RSc1723* for a protein secreted through the type III secretion system (T3SS) and type VI secretion system‐related genes, but also EPS I productivity‐related genes (Fig. 1A, Tables [Link], [Link] and [Link], [Link], see Supporting information). Furthermore, among the negatively EPS I‐regulated genes, 100 genes were included in the negatively *phc *QS‐regulated genes, including chemotaxis‐related genes, flagellar motility‐related genes, five T3SS‐related genes and six effector genes secreted through the T3SS (Fig. 1B, Tables [Link], [Link] and [Link], [Link], see Supporting information). Conversely, we detected no significant differences between Δ*epsB* and OE1‐1 in the expression levels of *phcB* and *phcA* (Table S1). The expression levels of all the transcripts in Δ*epsB* were positively correlated with those in Δ*phcB* (Fig. 1C) and Δ*phcA* (Fig. 1D). Although the expression levels of all the transcripts in Δ*epsB* were positively correlated with those in the ralfuranone productivity‐deficient mutant (Δ*ralA*; Kai *et al.*, 2014)*,* the coefficients of correlation were lower than those between Δ*epsB* and the *phc *QS‐deficient mutants (Fig. 1E).

**Figure 1:**
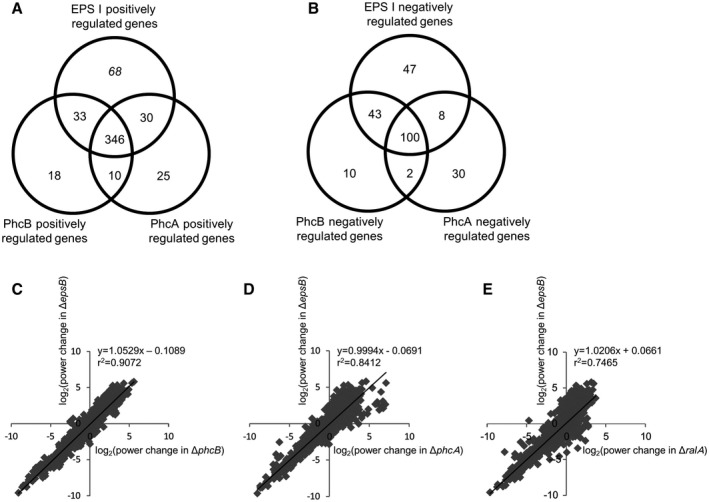
The numbers of genes that exhibited expression level fold‐changes ≤−4 (A) or ≥4 (B) in the *Ralstonia solanacearum phcB*‐deletion mutant (Δ*phcB*), *phcA*‐deletion mutant (Δ*phcA*) and *epsB*‐deletion mutant (Δ*epsB*), which lost major EPS I productivity, relative to their expression levels in strain OE1‐1, and correlations in gene expression level changes between Δ*phcB* and Δ*epsB* of *R. solanacearum* strains (C), between Δ*phcA* and Δ*epsB* of *R. solanacearum* strains (D), and between the ralfuranone productivity‐deficient mutant (Δ*ralA*) and Δ*epsB* of *R. solanacearum* strains (E). The fragments per kilobase of open reading frame per million fragments mapped values for *R. solanacearum* strains OE1‐1, Δ*phcB*, Δ*phcA,* Δ*ralA* and Δ*epsB* were normalized prior to analyses of differentially expressed genes. Data for the genes affected by the deletion of *ralA* are from Mori *et al*. (2018b).

We then created a native *epsB*‐expressing complemented Δ*epsB* mutant (*epsB*‐comp). An 803‐bp DNA fragment (cepsB‐1) of OE1‐1 genomic DNA was amplified by PCR using cepsB‐1‐FW (5′‐TGTCG AAGTT CATGT CCCAC ACC‐3′) and cepsB‐1‐RV (5′‐ATGCG ACCAC CATGA TCGGA TGTCC TTGCG TC‐3′) as primers. A 2322‐bp DNA fragment (cepsB‐2) of OE1‐1 genomic DNA was amplified by PCR using the primers cepsB‐2‐FW (5′‐GGACG CATTC ATGAC GCAGA ACCTC TCTCA GC‐3′) and cepsB‐2‐RV (5′‐CATTC CCCTC CTGAT TCGCA ATC‐3′). A 3104‐bp DNA fragment was then amplified by PCR using cepsB‐1 and cepsB‐2 as templates and cepsB‐1‐FW and cepsB‐2‐RV as primers. The 3104‐bp DNA fragment was ligated into pMD20 (Takara Bio, Otsu, Japan) to create pMD20cepsB (Table 1). A *Kpn*I‐ and *Spe*I‐digested 3.2‐kb fragment of pMD20cepsB was ligated into a *Kpn*I‐ and *Spe*I‐digested pUC18‐mini‐*Tn*7‐Gm (Choi *et al.*, 2006) to create pCepsB (Table 1). pCepsB was electroporated into Δ*epsB* with pTNS2 (Choi *et al.*, 2006), and the gentamycin‐resistant strain *epsB*‐comp was created.

**Table 1:** Strains and plasmids used in this study

	Relevant characteristics	Source
Plasmids used for cloning		
pMD20	pUC19 derivative, Amp^r^	Takara Bio
pUC18‐mini‐*Tn*7‐Gm	Gm^r^	Choi *et al. *(2006)
pTNS2	Helper plasmid carrying T7 transposase gene	Choi *et al. *(2006)
pMD20cepsB	pMD20 derivative carrying a 3.1‐kbp fragment containing *epsB*	This study
pCepsB	pUC18‐mini‐*Tn*7T‐Gm derivative carrying a 3.1‐kbp fragment containing *epsB*, Gm^r^	This study
*Escherichia coli*		
DH5α	*recA1 endA1 gyrA96 thi‐1 hsdR17supE44* Δ(*lac*)*U169*(ϕ*80lac*ΔM15)	Takara Bio
*Ralstonia solanacearum*		
OE1‐1	Wild‐type strain, phylotype I, race 1, biovar 4	Kanda *et al. *(2003)
Δ*epsB*	*epsB*‐deletion mutant of OE1–1	Mori *et al *(2018a)
*epsB*‐comp	A transformant of Δ*epsB* with pCepsB containing native *epsB*, Gm^r^	This study
Δ*phcB*	*phcB*‐deletion mutant of OE1–1	Kai *et al. *(2015)
Δ*phcA*	*phcA*‐deletion mutant of OE1–1	Mori *et al. *(2016)

The expression levels of *phcB* and *phcA* were analysed in *R. solanacearum* strains Δ*epsB*, *epsB*‐comp and OE1‐1 grown in 1/4 M63 (until OD_600_ = 0.3) by quantitative reverse transcription‐PCR (qRT‐PCR) assays. We conducted a qRT‐PCR assay using a 500 ng total RNA template sample and 10 pm primers (Table 2) with an Applied Biosystems 7300 Real‐time PCR system (Applied Biosystems, Foster City, CA, USA) as described by Mori *et al. *(2016). We normalized all the values against the *rpoD* expression level in which there was no significant difference among samples. We conducted the experiment twice using independent samples with eight technical replicates in each experiment. Because we did not observe any significant differences between replicates, we provided results of a single representative sample. We did not observe any significant differences in the expression levels of *phcB* and *phcA* among these strains (*P* < 0.05, Fig. 2).

**Table 2:** Primers used in the qRT‐PCR assays

Name of gene	Name of primer	Nucleotide sequence (5′–3′)
*rpoD*	rpoD‐FW	ATCGTCGAGCGCAACATCCC
	rpoD‐RV	AGATGGGAGTCGTCGTCGTCGTG
*fliC*	fliC‐FW2	CAAACGCAAGGTATTCAGAACG′
	fliC‐RV2	ATTGGAAGGTCGTCGAAGCCAC
*lecM*	fml‐FW2	GTATTCACGCTTCCCGCCAACAC
	fml‐RV2	ATGCCGTCGTTGTAGTCGT TGTC
*phcB*	phcB‐FW3‐514	TACAAGATCAAGCACTACCTCGACTG
	phcB‐RV3‐1011	GTGCTGTACGCCATCCATCTC
*phcA*	phcA‐FW5	ATGCGTTCCAATGAGCTGGAC
	phcA‐RV5	AGATCCTTCATCAGCGAGTTGAC
*ralA*	ralA‐FW	GCCTGGGGATAAGGTTGTAC
	ralA‐RV	CGTCAGTACGAAAACAGCG

**Figure 2:**
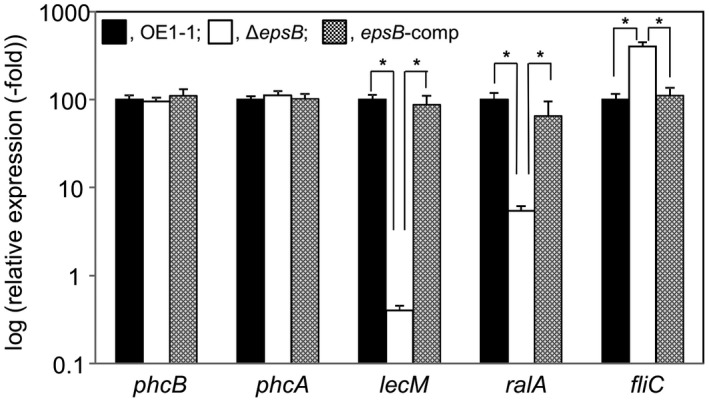
Expression of the quorum sensing (*phc *QS)‐related genes *phcB* and *phcA*, the positively *phc *QS‐regulated genes *lecM* and *ralA*, and the negatively *phc *QS‐regulated gene *fliC* in strains OE1‐1, the *epsB*‐deletion mutant (Δ*epsB*), which lost major EPS I productivity, and native *epsB*‐expressing complemented Δ*epsB* (*epsB*‐comp) of *Ralstonia solanacearum*. Total RNA was extracted from the bacterial cells grown in one‐quarter‐strength M63 medium (until OD_600_ = 0.3). The gene expression levels were presented relative to the *rpoD* expression level. The experiment was conducted twice using independent samples with eight technical replicates in each experiment, and produced similar results. Results of a single representative sample are provided. Bars indicate the standard errors. Asterisks indicate values that are significantly different between strains (*P* < 0.05, *t*‐test).

The transcriptome analysis showed that deletion of *epsB* led to significantly reduced expression levels of *ralA* and *ralD*. We next assessed the production of ralfuranones by *R. solanacearum* strains Δ*epsB*, *epsB*‐comp and OE1‐1 that were grown in 100 mL of MGRL medium (1.75 mm sodium phosphate buffer, pH 5.8: 1.5 mM MgSO_4_: 2.0 mM Ca(NO_3_)_2_: 3.0 mM KNO_3_: 67 Mm Na_2_EDTA: 8.6 Mm FeSO_4_: 10.3 Mm MnSO_4_: 30 Mm H_3_BO_3_: 1.0 Mm ZnSO_4_: 24 nM (NH_4_)_6_Mo_7_0_24_: 130 nM CoC1_2_: 1 μM CuS0_4_; Fujiwara *et al.*, 1992) including 3% sucrose for 4 days, according to a previously described procedure (Kai *et al.*, 2014). Δ*epsB* produced less ralfuranones A, B, J, K and L than the strain OE1‐1 as well as *epsB*‐comp strain (Fig. 3). To analyse the *ralA* expression levels, we conducted a qRT‐PCR assay using 10 pm primers (Table 2). We observed a significantly lower expression level of *ralA* in Δ*epsB* than in the strain OE1‐1 as well as *epsB*‐comp strain (*P* < 0.05, Fig. 2). Therefore, deletion of *epsB* led to significantly reduced production of ralfuranones.

**Figure 3:**
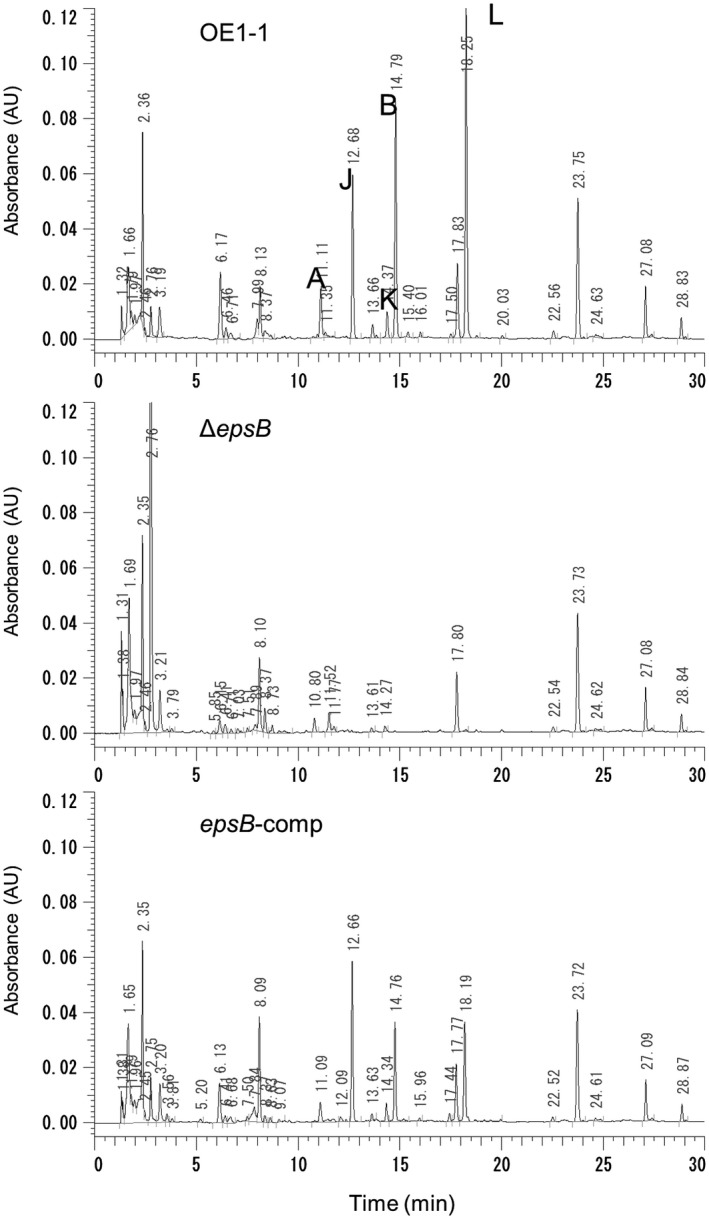
Ralfuranone productivity of the OE1‐1 (A), the *epsB*‐deletion mutant (Δ*epsB*, B), which lost major EPS I productivity, and native *epsB‐*expressing complemented Δ*epsB* (*epsB*‐comp, C) *Ralstonia solanacearum* strains. An HPLC analysis of culture extracts from the *R. solanacearum* strains is shown. The peaks of ralfuranones are marked A, B, J, K and L. The experiment was conducted at least twice using independent samples and produced similar results. Results for a single representative sample are provided.

The transcriptome analysis showed that deletion of *epsB* led to significantly reduced expression levels of *lecM*. LecM affects the extracellular stability of 3‐OH MAME, contributing to the *phc *QS signalling pathway (Hayashi *et al.*, 2019). We next assayed the extracellular 3‐OH MAME contents of *R. solanacearum* strains Δ*epsB*, *epsB*‐comp and OE1‐1. We assessed the extracellular 3‐OH MAME contents of *R. solanacearum* strains that were grown in B medium at 30 °C for 4–6 h, according to a previously described protocol (Kai *et al.*, 2015). We observed a lower extracellular 3‐OH MAME content for Δ*epsB* than for the OE1‐1 and *epsB*‐comp strains (*P* < 0.05, Fig. 4A). To analyse the *lecM* expression levels in *R. solanacearum* strains, we conducted a qRT‐PCR assay with 10 pm primers described in Table 2. The expression level of *lecM* in Δ*epsB* was significantly lower than those in the strain OE1‐1 as well as *epsB*‐comp strain (*P* < 0.05, Fig. 2). Because the deletion of *epsB* did not change the expression of *phcB*, it was thought that the deletion of *epsB* resulted in significantly reduced *phc *QS‐dependent *lecM* expression, affecting the extracellular stability of 3‐OH MAME.

**Figure 4:**
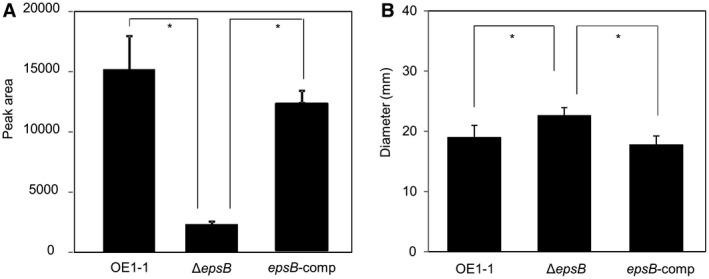
The extracellular content of 3‐OH MAME (A) and swimming motility (B) of *Ralstonia solanacearum* strains OE1‐1, the *epsB*‐deletion mutant (Δ*epsB*), which lost major EPS I productivity, and native *epsB‐*expressing complemented Δ*epsB* (*epsB*‐comp). (A) The experiment was conducted three times using independent samples. Bars indicate the standard errors. Asterisks indicate values significantly different from those of strain OE1‐1 (*P* < 0.05, *t*‐test). (B) The experiment was repeated three times, with five technical replicates in each experiment. Bars indicate the standard errors. Asterisks indicate values that are significantly different between strains (*P* < 0.05, *t*‐test).

The *phc *QS negatively regulates flagellar biogenesis, suppressing the swimming motility of *R. solanacearum* (Tans‐Kersten *et al.*, 2001)*.* To analyse the expression levels of *fliC* encoding flagellin in the strains, we conducted qRT‐PCR assays using 10 pm primers (Table 2). We observed a significantly greater expression level of *fliC* in Δ*epsB* than in the strain OE1‐1 as well as *epsB*‐comp strain (*P* < 0.05, Fig. 2). We next analysed the swimming motilities of the Δ*epsB*, *epsB*‐comp and OE1‐1 strains incubated on 1/4 M63 solidified with 0.25% agar. Δ*epsB* strain exhibited significantly greater swimming motility than the OE1‐1 strain, which was similar to that of the *epsB*‐comp mutant (*P* < 0.05, Fig. 4B).

A transcriptome analysis with RNA‐Seq showed that a loss of EPS I productivity led to changes in the expression levels of *c*.15% of the genes expressed in strain OE1‐1 (Fig. 1). Among them, the expression levels of the genes that are positively and negatively regulated through the *phc *QS in strain OE1‐1 decreased (97.2%; Fig. 1A) and increased (98.0%; Fig. 1B), respectively, in Δ*epsB*. The expression levels of genes in Δ*epsB* were positively correlated with those in both Δ*phcB* (Fig. 1C) and Δ*phcA* strains (Fig. 1D). Furthermore, a deficiency of EPS I productivity led to significantly reduced ralfuranone production (Fig. 3) and a significantly enhanced swimming motility (Fig. 4B). Furthermore, the deficiency of EPS I productivity led to a significantly reduced *lecM* expression (Tables S1 and Fig. 2) and extracellular 3‐OH MAME content (Fig. 4A). Thus, the deficiency of EPS I production induces a negative feedback effect on the regulation of PhcA‐controlled genes, contributing to *phc *QS‐dependent phenotypes. Overall, EPS I may be associated with the feedback loop of QS (Fig. S1).

The *epsB* deletion did not affect the expression of *phcB* and *phcA* (Fig. 2 and Table S1). Although a loss in EPS I productivity led to the increased expression of 477 genes and the decreased expression of 198 genes that were expressed in strain OE1‐1, the expression levels of 27.5% and 49.5% of these genes, respectively, were independent of *phc *QS (Fig. 1). Thus, EPS I may not directly affect *phcA* expression and PhcA function, but may be involved in mechanisms supporting regulation through PhcA or a co‐transcription activator functioning with PhcA. Although the expression levels of all the transcripts in Δ*epsB* were positively correlated with those in Δ*ralA* (Fig. 1E), the coefficients of correlation were lower than those of the expression levels of all the transcripts between Δ*epsB* and the *phc *QS‐deficient mutants (Fig. 1C,D). Thus, EPS I‐supported mechanisms of PhcA function may be different from the ralfuranone‐mediated feedback of *phc *QS.

In this study, the deficiency of EPS I production led to an induction of a negative feedback effect on the regulation of *phc *QS‐dependent genes. Additionally, 85% of EPS I is released as a cell‐free slime (Denny, 2006). Therefore, EPS I may mediate intercellular signalling among OE1‐1 cells, contributing to the regulation of PhcA‐controlled genes during *phc *QS.

## Supporting information


**Fig. S1** Model of the regulation of quorum sensing (*phc *QS) in *Ralstonia solanacearum* strain OE1‐1.

**Table S1** RNA‐sequencing data for all the transcripts in *Ralstonia solanacearum* strain OE1‐1, *phcB*‐deletion mutant (Δ*phcB*), *phcA*‐deletion mutant (Δ*phcA*) and *epsB*‐deletion mutant (Δ*epsB*), which lost major exopolysaccharide (EPS I) productivity.

**Table S2** Predicted functions of proteins encoded by genes that were both positively major exopolysaccharide (EPS) I‐regulated and positively *phc *QS‐regulated in *Ralstonia solanacearum* strain OE1‐1 grown in one‐quarter‐strength M63 medium.

**Table S3** Predicted functions of proteins encoded by genes that were negatively major exopolysaccharide (EPS) I‐regulated and negatively *phc *QS‐regulated in *Ralstonia solanacearum* strain OE1‐1.
