# Supplementary figures and images for: Major exopolysaccharide, EPS I, is associated with the feedback loop in the quorum sensing of Ralstonia solanacearum strain OE1‐1
Source: Mol Plant Pathol. 2019 Sep 27;20(12):1740–7. doi: 10.1111/mpp.12870 (PMC6859485; doi:10.1111/mpp.12870)

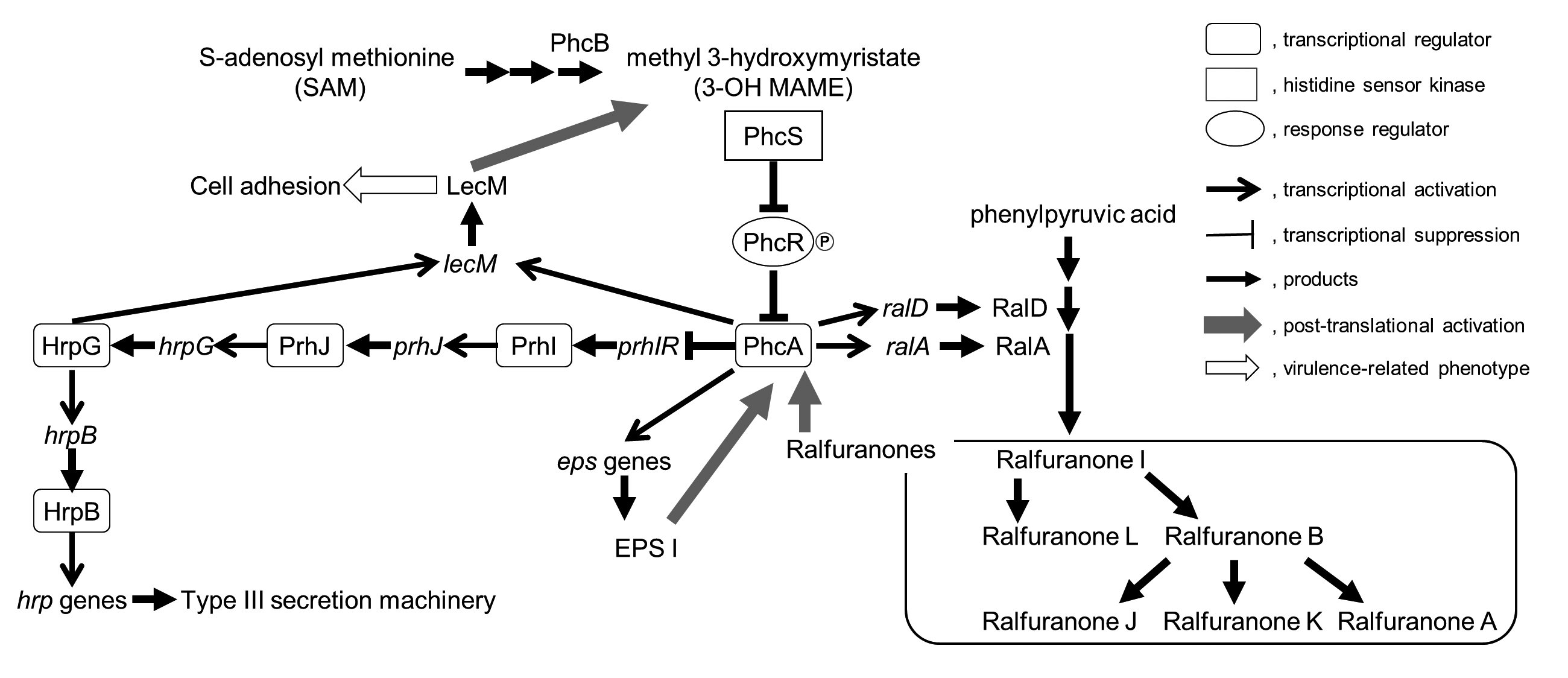

Supplement: Supplementary file 1 — Fig. S1 Model of the regulation of quorum sensing (phc QS) in Ralstonia solanacearum strain OE1‐1. [file MPP-20-1740-s001.tif]
